# Comparison of Osteosarcoma Aggregated Tumour Models with Human Tissue by Multimodal Mass Spectrometry Imaging

**DOI:** 10.3390/metabo11080506

**Published:** 2021-07-31

**Authors:** Lucy E. Flint, Gregory Hamm, Joseph D. Ready, Stephanie Ling, Catherine J. Duckett, Neil A. Cross, Laura M. Cole, David P. Smith, Richard J. A. Goodwin, Malcolm R. Clench

**Affiliations:** 1 Centre for Mass Spectrometry Imaging, Biomolecular Research Centre, Sheffield Hallam University, Sheffield S1 1WB, UK; lf8183@exchange.shu.ac.uk (L.E.F.); jr8158@exchange.shu.ac.uk (J.D.R.); c.duckett@shu.ac.uk (C.J.D.); n.cross@shu.ac.uk (N.A.C.); l.cole@shu.ac.uk (L.M.C.); d.p.smith@shu.ac.uk (D.P.S.); 2 Imaging and Data Analytics, Clinical Pharmacology and Safety Sciences, BioPharmaceuticals R&D, AstraZeneca, Cambridge CB4 0WG, UK; gregory.hamm@astrazeneca.com (G.H.); stephanie.ling@astrazeneca.com (S.L.); richard.goodwin@astrazeneca.com (R.J.A.G.); 3 Institute of Infection, Immunity and Inflammation, College of Medical, Veterinary and Life Sciences, University of Glasgow, Glasgow G12 8QQ, UK

**Keywords:** osteosarcoma, DESI, mass spectrometry imaging, LA-ICP-MS, metabolomics, imaging mass cytometry

## Abstract

Osteosarcoma (OS) is the most common primary bone malignancy and largely effects adolescents and young adults, with 60% of patients under the age of 25. There are multiple cell models of OS described in vitro that express the specific genetic alterations of the sarcoma. In the work reported here, multiple mass spectrometry imaging (MSI) modalities were employed to characterise two aggregated cellular models of OS models formed using the MG63 and SAOS-2 cell lines. Phenotyping of the metabolite activity within the two OS aggregoid models was achieved and a comparison of the metabolite data with OS human tissue samples revealed relevant fatty acid and phospholipid markers. Although, annotations of these species require MS/MS analysis for confident identification of the metabolites. From the putative assignments however, it was suggested that the MG63 aggregoids are an aggressive tumour model that exhibited metastatic-like potential. Alternatively, the SAOS-2 aggregoids are more mature osteoblast-like phenotype that expressed characteristics of cellular differentiation and bone development. It was determined the two OS aggregoid models shared similarities of metabolic behaviour with different regions of OS human tissues, specifically of the higher metastatic grade.

## 1. Introduction

Osteosarcoma (OS) is the most common primary bone malignancy and largely effects adolescents and young adults, with 60% of patients under the age of 25. Bone sarcomas are classified as a rare type of cancer with OS having an estimated incidence of approximately 7 in 100,000 persons, with 30,000 new cases a year [1]. Despite improvements in treatment therapies over the recent years, the survival rate of bone sarcomas has remained unchanged with <50% at 5 years [2]. The aggressive tumoral behaviour of OS has challenged the development of an effective therapeutic treatment. Even with complete surgical resection of the primary tumour, approximately 90% of OS patients develop metastasis such as of the lung or breast [3]. Hence, there is still a great need for studying the molecular activity within the sarcoma in order to understand the metastatic behaviour and improve treatment therapies.

There are multiple cell models of OS described in vitro that express the specific genetic alterations of the sarcoma. The most common OS cell lines include the epithelial-like cells, SAOS-2 and the fibroblastic-like cells, MG63, both with a deficiency mutation in the tumour suppressor p53 gene. However, significant differences in the phenotypes of these cell lines have contributed to inconsistent results across studies. The SAOS-2 cells express a mature osteoblast phenotype with a high level of alkaline phosphatase (ALP) activity [4] a gene associated with bone mineralisation. In contrast, the MG63 cell line represent the immature osteoblast phenotype with low ALP activity and matrix differentiation potential [5].

With differences in phenotypes, the expression of endogenous molecules would also be expected to vary, and thus impact the responses to treatment. Multiple previous studies have characterised the OS cell lines by DNA profiling and the expression of proteins using conventional methods, such as PCR analysis and immunostaining. For example, the detection of metastatic-related OS gene expressions, such as PHLDA1 enabled the categorisation of 18 OS cell lines from high to low metastatic potential, marking SAOS-2 and MG63 as relatively low metastatic cell lines [3]. In addition, heterogenous expressions of ECM proteins such as collagen I and III, and MMP-9 were used to differentiated SAOS-2 cells from MG63 cells [6]. Untargeted liquid chromatography tandem mass spectrometry (LC-MS/MS) approaches have also been employed in metabolomics studies of both OS cell lines [7,8]. The limitations of these studies, however, is the characterisation of the OS cell lines in 2D, which is not representative of in vivo behaviour. 

Alternatively, three-dimensional (3D) cell cultures, such as tumour spheroids, offer a biologically relevant model that can be used for cancer research and early-stage drug development studies. The cellular complexity of 3D models mimics tumour microenvironment of proliferative, hypoxic, and necrotic regions through gradients of oxygen and nutrients. By recapitulating the cell−cell interactions and the gene and protein expressions of tumours in a way that 2D cultures cannot, 3D cultures are therefore a valuable research tool to study realistic drug behaviour. For instance, significant changes in the metabolite levels of lactate and alanine between 2D and 3D MG63 cultures were detected by high resolution nuclear magnetic resonance (NMR) [9]. Additionally, alterations in the proteome between monolayer and spheroid cultures of canine OS cells by differential gel electrophoresis (DIGE) matrix-assisted laser desorption ionisation (MALDI)-MS has also been reported [10]. However, according to the literature the characterisation of OS 3D culture models has still been limited to profiling or immunostaining techniques, which leads to loss of spatial information and/or requires target-specific analysis [10,11,12].

In order to understand the wide molecular activity that drives OS and captures the heterogeneity of the tissue, a comprehensive analysis strategy is required. Mass spectrometry imaging (MSI) is a multiplex methodology that has the capability to map molecular distributions within biological tissues via an unlabelled approach. The spatial localisation of a molecule can determine the interplay of biological functions and interactions within a tissue. This also enables a greater biological understanding of cellular phenotypes and their structural organisation, in addition to the surrounding microenvironment. For example, previously defined proliferative and apoptotic and necrotic regions within a SAOS-2 spheroid aggregate model has been determined by detecting heterogeneous distributions of endogenous metabolites using MALDI-MSI [13]. Furthermore, we have previously employed multimodal MSI techniques to demonstrate how obtaining a large amount of molecular information with a complementary nature can enhance the understanding of the biological processes within a tissue. The detection of metabolites, proteins and metals determined the interplay between molecules and established key pathways that defined a novel HCC827 lung adenocarcinoma ‘aggregoid’ model [14]. A similar approach could therefore give a valuable insight into 3D tumour aggregoid models of OS, providing comprehensive information of biochemical pathways that influence the cancer pathogenesis. In addition to this, a recent paper demonstrated the combination of oesophageal cancer multicellular tumour spheroids (MCTS) and clinical tissue with MALDI-MSI as an approach to determine the metabolic relevance of the models to in vivo [15]. With that in mind, detailed molecular characterisation of in vitro OS models and clinical tissue would improve the understanding of cancer metabolism within the 3D cultures and highlight biomarkers of patient outcome. 

In the work reported here, multiple MSI modalities were employed to characterise two novel OS aggregoid models, MG63 and SAOS-2, for the purposes of developing a potential in vitro methodology for pre-clinical cancer research and drug development. Firstly, the metabolic profiles of the models were determined by desorption electrospray ionisation (DESI)-MSI and compared against the established tumour microenvironment of the HCC827 lung adenocarcinoma aggregoid. An investigation into the relevance of the OS aggregoid models with clinical OS patient samples was then conducted to determine the similarities in the metabolite expressions that influenced tumour behaviour and potentially detect metastatic-like activity. In addition, imaging mass cytometry (IMC) and laser ablation inductively coupled plasma (LA-ICP)-MSI were utilised to further establish the phenotypical characteristics of the tumour microenvironments of both aggregoid models through protein and protein modification markers and metal compositions, respectively.

## 2. Results

### 2.1. Metabolite Imaging

#### 2.1.1. Aggregoid Phenotyping

To determine the metabolic profiles of the two OS aggregoid models, a comparative analysis was performed by employing DESI-MSI. In this study, the metabolic activity within three biological replicates of the MG63 and SAOS-2 aggregoid models were determined. Metabolite data of a HCC827 lung adenocarcinoma model were also included as a reference to understand the phenotypes within the OS models.

**Figure 1:**
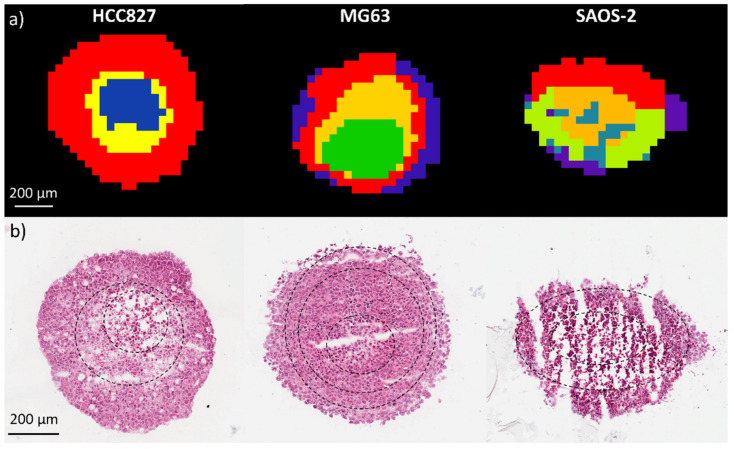
Spatial segmentation of a HCC827, MG63 and SAOS-2 aggregoid from metabolite data by DESI-MSI. (**a**) Spatial segmentation identified heterogeneous clustering phenotypes between aggregoid models. Segmentation of each sample was performed independently and therefore the coloured clusters do not correspond between samples. (**b**) H&E stain of same aggregoid sections from each model. Black dotted line highlights the approximate phenotypical regions identified by segmentation clustering analysis. Scale bar 200 μm.

Initially, the data were spatially segmented to determine phenotypical regions within the aggregoid sections. From the segmentation analysis, differences of the phenotypical regions in each model were observed (Figure 1). As reported previously [14], the segmented image of an HCC827 aggregoid model comprised three regions: a distinctive inner core, an annular zone and an outer region, which were also observable in the histological image. The segmentation of the MG63 model showed a similar pattern, where a core, two annular regions and a periphery were observable in the segmented MSI data. From the histology however, the MG63 model differed from the HCC827 aggregoid in that the cells were tightly compacted throughout the section and although some condensing of DNA was observed, no significant apoptotic bodies were identified. This suggests that the core cluster within the MG63 aggregoid does not necessarily correspond to an obvious region of cellular stress due to hypoxia. Yet, the segmentation analysis was still able to identify changes in metabolite activity affected by the lack of oxygen and nutrients towards the core of the aggregoid.

The SAOS-2 model showed significantly different features to the HCC827 and MG63 models in the segmentation analysis (Figure 1). Although a core was identified, the clustering pattern showed a lot of heterogeneity across the section, which was similar in each SAOS-2 sample (*n* = 3). Examination of the histological data indicated that a lot of fracturing was present. This has been noted across the SAOS-2 aggregoid samples throughout the experimental work conducted and is potentially due to the nature of culturing the SAOS-2 cell line. A previous study reported a heterogenous capability to form 3D cultures between OS cell lines and noted that the SAOS-2 cells formed irregular spheroids, whereas the MG63 formed more spherical spheroid [16]. Interestingly, from the histology data in the paper the SAOS-2 spheroids displayed a compact cellular distribution. However, the spheroids were significantly smaller (≤200 μm diameter) in comparison to the aggregoid model reported here (~1 mm diameter). The histology data in this study showed that a large area of apoptotic and necrotic activity within the SAOS-2 aggregoid was present. This is likely impacting the stability of the aggregoids. As this has been observed throughout the SAOS-2 samples, it is most likely due to the morphological nature of the cell line. Nevertheless, phenotypical regions within the SAOS-2 model were still observed and metabolite distributions were detected.

**Figure 2:**
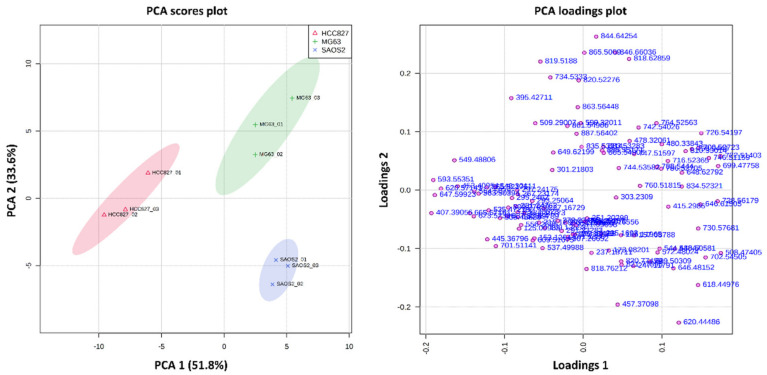
PCA scores and loadings plot show separation of the MG63 and SAOS-2 aggregoid models from the HCC827 aggregoid model. Principal components, PC 1 (51.8%) and PC 2 (33.6%) showed the best separation between sample groups. The discriminatory *m*/*z* values of interest were distributed separately from the cluster of peaks. Samples were grouped with 95% confidence, HCC827 (red), MG63 (green), and SAOS-2 (blue).

**Figure 3:**
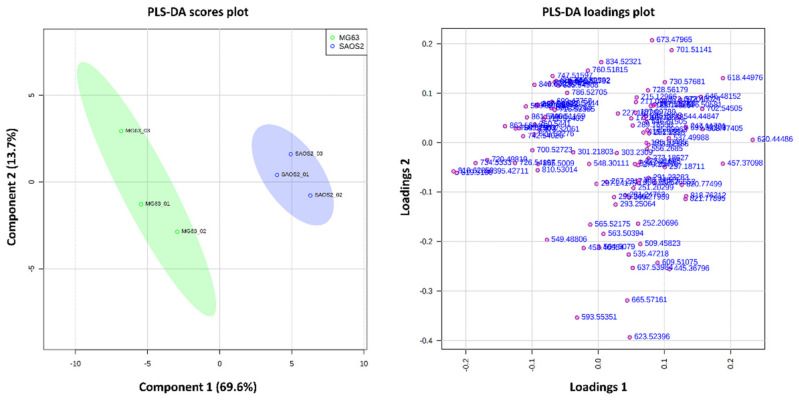
PLS-DA scores and loadings plot show variance between MG63 aggregoid model and the SAOS-2 aggregoid model. Component 1 (69.6%) and component 2 (13.7%) showed the best separation between samples. The discriminatory *m*/*z* values of interest were distributed separately from the cluster of peaks. Samples were grouped with 95% confidence, MG63 (green) and SAOS-2 (blue).

PCA was conducted to determine the differences between the three aggregoid models. From the analysis, an obvious separation between the cell lines was observed with relatively tight 95% confidence grouping (Figure 2). The distribution of the OS models within the scores plot suggested more similarities in the metabolites compared to the lung model. However, from the PLS-DA, there was clear variance in the detected metabolites between the MG63 and SAOS-2 aggregoid models (Figure 3). This is as expected, as the fibroblastic MG63 and epithelial SAOS-2 cell types will exhibit different metabolic behaviour. A summary of the key metabolites identified in the aggregoid models is reported in Table 1. A significant number of metabolites including fatty acids and lipids were detected and are discussed.

**Figure 4:**
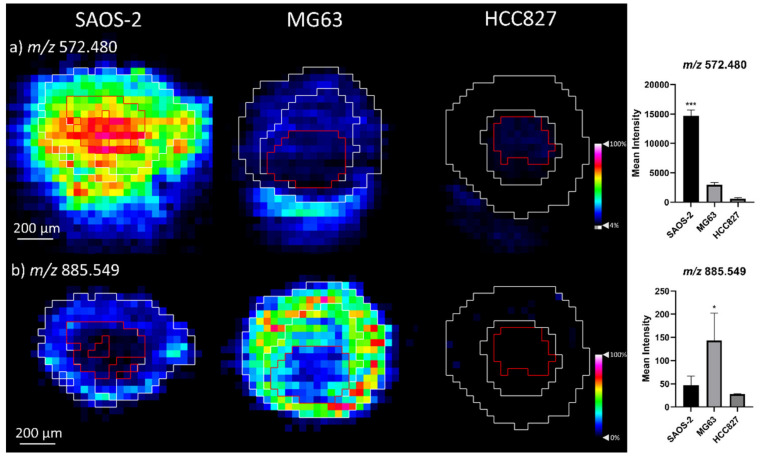
Distribution of metabolites significantly detected in OS aggregoid models. Ion density maps of metabolites outlining the core and outer area on the image. Mean intensity plotted on bar graph against the SAOS-2, MG63 and HCC827 aggregoids. Data is mean ± SD (*n* = 3), one-way ANOVA with Tukey post hoc test * *p* < 0.05, *** *p* < 0.001. Scale bar 200 μm. Peaks identified (**a**) *m*/*z* 572.480, Cer 34:1; O2 [M + Cl]^−^; (**b**) *m*/*z* 885.549, PI 38:4.

From the observed metabolites, differences in the detection levels across the tumour aggregoid models were observed. In particular, ceramide species Cer 32:1; O2, Cer 34:1; O2, and their chlorine adducts were significantly elevated in the SAOS-2 model (Table 1). From the image, the intensity of Cer 34:1; O2 [M + Cl]^−^ at *m*/*z* 572.480 was localised across the aggregoid with concentrated levels within the core (Figure 4a). Some detection was observed in the MG63 aggregoid, and even less so in the HCC827 model. Within the MG63 aggregoid models, glycerophospholipid species, including phosphatidylcholine (PC), phosphatidylethanolamine (PE), phosphatidylserine (PS) and phosphatidylinositol (PI) were expressed considerably higher in comparison to the SAOS-2 and HCC827 models (Table 1). 

#### 2.1.2. Comparison of OS Aggregoid Models with OS Human Tissue

**Figure 5:**
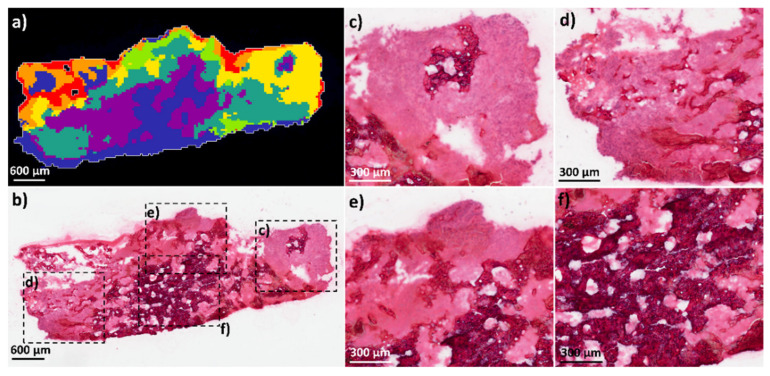
Spatial segmentation analysis of OS patient_826 tissue sample from metabolite data by DESI-MSI. (**a**) spatial segmentation of OS section highlighting heterogeneous clusters throughout tissue. Scale bar 600 μm. (**b**) H&E of same OS tissue section after image analysis. Scale bar 600 μm. Magnification of ROIs: (**c**) solid tumour (corresponding to the yellow cluster) with osteoid island (corresponding to the purple/blue clusters) at the right of the tissue; (**d**) region of tumour and mineralised bone located to the left of tissue (corresponding to the yellow and sage green clusters); (**e**) tumour and mineralised bone located at the top of the tissue (corresponding to the sage green cluster); (**f**) Dense osteoid bone present focally (purple/blue cluster). Scale bar 300 μm.

The metabolic data obtained from the OS human tissue samples was spatially segmented to classify the clusters with specific phenotypic regions. The spatial segmentation analysis showed complex clustering patterns within all tissue sections in comparison to the aggregoid models. This is due to the heterogeneity of the tissues comprising of many cell types that form the tumorous tissue in addition to bone and cartilage. Figure 5a shows the spatial segmentation of a tissue section from OS patient_826 imaged by DESI-MSI and the histology stain after analysis (whole H&E image Figure 5b and enlarged regions Figure 5c–f). From the pathology report, it was determined that the specific sample had cancerous tumour regions identified throughout the tissue and the osteoid bone was formed by tumour cells. Therefore, detection of different tumorous phenotypes throughout the whole tissue section was expected. From the segmentation analysis, a solid tumour region (yellow cluster) with an osteoid bone island (purple/blue clusters) was identified on the right of the tissue (Figure 5c). Similarities to this yellow tumour cluster were also detected throughout the sample (Figure 5d). A large area of mineralised bone within tumorous tissue was also detected and determined by the sage green cluster (Figure 5d,e). In addition, a significant area of dense osteoid bone with a filagree pattern was focally present and outlined by purple/blue clusters (Figure 5f).

**Figure 6:**
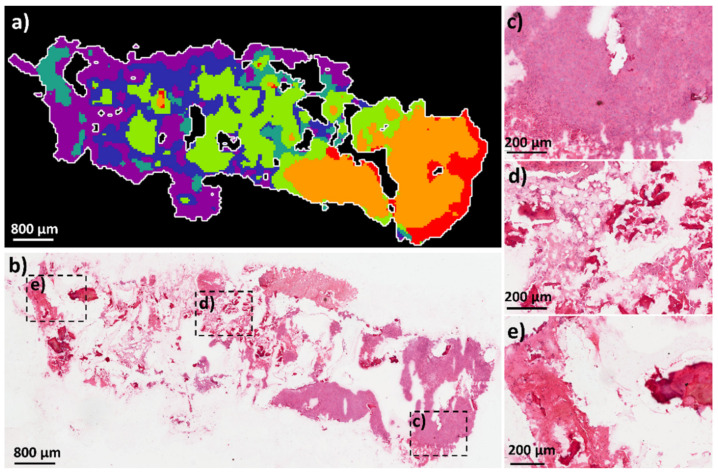
Spatial segmentation analysis of OS patient_882 tissue sample from metabolite data by DESI-MSI. (**a**) spatial segmentation of OS section highlighting heterogeneous clusters throughout tissue. Scale bar 800 μm. (**b**) H&E of same OS tissue section after image analysis. Scale bar 800 μm. Magnification of ROIs in H&E image: (**c**) solid tumour (corresponding to the orange cluster) at the right of the tissue; (**d**) region of medullary space with osteoid bone located focally (corresponding to the green and blue clusters); (**e**) lamellar bone located at the left of the tissue (corresponding to the sage green cluster). Scale bar 200 μm.

Figure 6 shows the spatial segmentation analysis of a tissue section from OS patient_882 and the histological image. From the pathology report, typical osteoblastic osteosarcoma phenotypes were detected. Regions of lamellar bone were identified, the medullary space contained various cell types with irregular nuclei, and osteoid production by atypical mitotic cells was determined. From the DESI data, the different phenotypes could be identified by the segmentation analysis. A large region of solid tumour was identified on the right of the tissue (orange cluster) (corresponding enlarged H&E image Figure 6c). The medullary space (blue cluster) with osteoid regions (green cluster) was localised within the focal region of the tissue (corresponding enlarged H&E image Figure 6d). A region of lamellar bone (sage green) and a large area of osteoid formation were localised within the left of the tissue (corresponding enlarged H&E image Figure 6e). It is noted that the segmentation analysis was performed independently for each patient sample and therefore the colour clusters do not correspond between groups. The segmentation analysis is based on the metabolite distribution, some areas of sample that show no tissue present on the histology images may show segmentation clusters due to the weak denoising of the image. Therefore, the segmentation analysis here gives a generalization of the area in complicated tissues such as the OS human patient samples.

**Figure 7:**
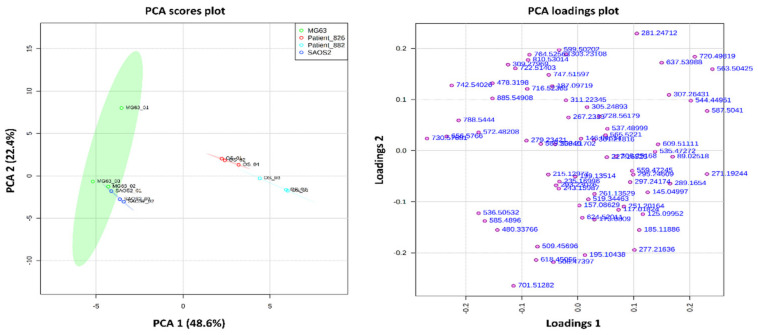
PCA scores and loadings plot show separation of the OS aggregoid models from the OS human tissue samples. Principle components, PC 1 (48.6%) and PC 2 (22.4%) showed the best separation between sample groups. The discriminatory *m*/*z* values of interest were distributed separately from the cluster of peaks. Samples were grouped with 95% confidence, MG63 (green), and SAOS-2 (blue), OS patient_826 (red) and OS patient_882 (light blue).

PCA was performed to determine the similarities and variances between the two OS aggregoid models with the two OS human tissue samples. It is stressed that the aggregoid data were acquired independently from the patient samples. Therefore, an indirect comparison has been performed in this study and thus the results are of a preliminary nature only. From the PCA scores plot, a clear separation of the aggregoid models from the OS human tissue samples was observed (Figure 7). As the human tissue is comprised of many heterogeneous regions and contains different cell types it was expected to have variance from the single-cell type aggregoid models. In addition, slight separation of the 95% confidence grouping of the two patient samples is also likely due to the heterogeneity between the tumour tissues. The MG63 aggregoid samples were relatively widely distributed which suggests some variability within the group, which could be due to slight culturing variation between replicates. For each remaining sample type however, tight clustering between the samples was observed. A summary of the key metabolites within the OS patient samples and the OS aggregoid models is reported in Table 2. The significant metabolites detected within the samples highlighted the similarities that exist between the OS aggregoid models and the OS human tissue.

**Figure 8:**
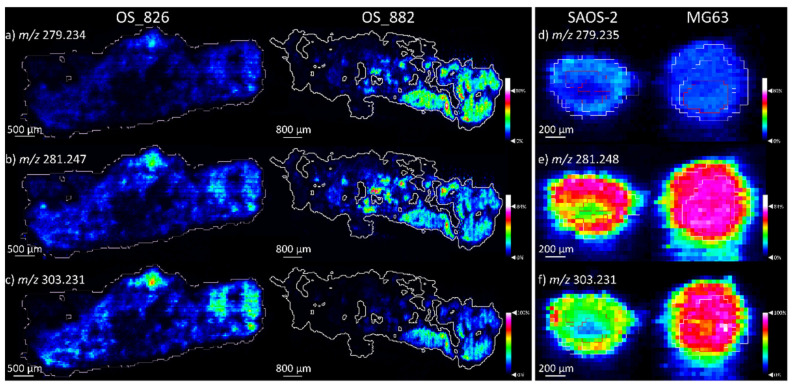
Fatty acid detection within OS human tissue and OS aggregoid models. Ion density maps of metabolites in OS patient_826 and OS patient_882. Scale bar 500 μm and 800 μm, respectively. Ion density maps of aggregoid models outlining the core and outer area. Scale bar 200 μm. Peaks identified in human tissue (**a**) *m*/*z* 279.234, FA 18:2; (**b**) *m*/*z* 281.247, FA 18:1; (**c**) *m*/*z* 303.231, FA 20:4. Peaks identified in OS models (**d**) *m*/*z* 279.235, FA 18:2; (**e**) *m*/*z* 281.248, FA 18:1; (**f**) *m*/*z* 303.231, FA 20:4. Mean intensity bar graph for each included in Supplementary Figure S1.

The major similarities between the OS human tissue samples and the OS aggregoid models is the detection of fatty acid species, specifically related to the arachidonic metabolism pathway. Figure 8a–c shows FA 18:2, e.g., linoleic acid at *m*/*z* 279.234, FA 18:1, e.g., oleic acid at *m*/*z* 281.247 and FA 20:4, e.g., arachidonic acid at *m*/*z* 303.231 within the OS human tissues (mean intensities included in Supplementary Figure S1a–c). The localisations of the metabolites were concentrated around the solid tumour regions of the OS human tissues and distributed throughout the similar tumorous areas as identified by the clustering analysis. These species were also identified at high levels in SAOS-2 and MG63 aggregoid models (Figure 8e,f and Supplementary Figure S1d,e). Within the SAOS-2 model, the metabolites were distributed more within the periphery, compared to the MG63 model where the species were heavily distributed throughout the section.

**Figure 9:**
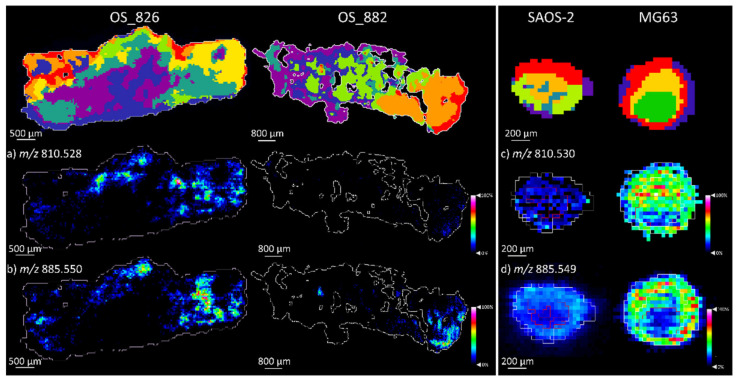
Phospholipid species detected within OS human tissue and OS aggregoid models. Ion density maps of metabolites in OS patient_826 and OS patient_882. Scale bar 500 μm and 800 μm, respectively. Ion density maps of aggregoid models outlining the core and outer area. Scale bar 200 μm. Peaks identified in human tissue (**a**) *m*/*z* 810.528, PS 38:4; (**b**) *m*/*z* 885.550, PI 38:4. Peaks identified in OS models (**c**) *m*/*z* 810.530, PS 38:4; (**d**) *m*/*z* 885.549, PI 38:4. Mean intensity bar graph for each included in Supplementary Figure S2.

The phospholipids PS 38:4 at *m*/*z* 810.528 and PI 34:4 at *m*/*z* 885.550, that were detected within the MG63 aggregoid were also detected significantly higher within the metastatic OS patient_826 tissues. Both species were observed within the large tumorous regions identified by the yellow and sage green cluster of OS patient_826, as low amounts of *m*/*z* 810.528 and *m*/*z* 885.550 were distributed in the orange tumour cluster of OS patient_882 (Figure 9 and Supplementary Figure S2).

**Figure 10:**
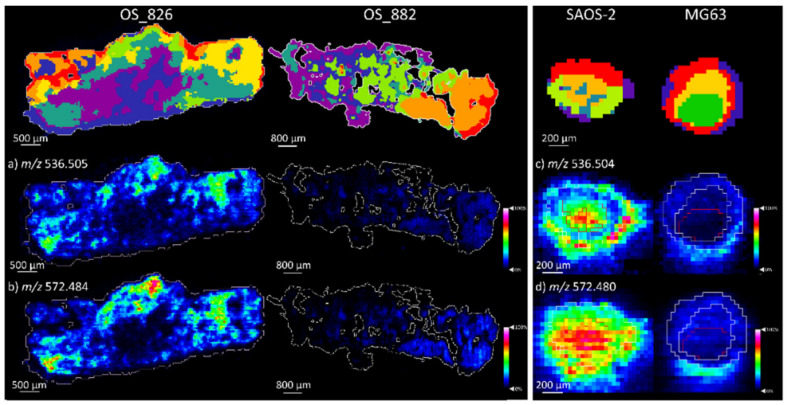
Ceramide species detected within OS human tissue and OS aggregoid models. Ion density scheme 826. and OS patient_882. Scale bar: 500 μm and 800 μm, respectively. Ion density maps of aggregoid models outlining the core and outer area. Scale bar: 200 μm. Peaks identified is human tissue a) *m*/*z* 536.505, Cer 34:1; O2; (**b**) *m*/*z* 572.484, Cer 34:1; O2 [M + Cl]^−^. Peaks identified in OS models (**c**) *m*/*z* 536.504, Cer 34:1; O2; (**d**) *m*/*z* 572.480, Cer 34:1; O2 [M + Cl]^−^. Mean intensity bar graph for each included in Supplementary Figure S3.

The ceramide species that defined the SAOS-2 aggregoid model were significantly expressed within the metastatic OS patient_826 tissues. Cer 34:1; O2 at *m*/*z* 536.505 and its chlorine adduct at *m*/*z* 572.484 [M + Cl]^−^ were localised within the tumorous regions that corresponded mainly to the sage green cluster of OS patient_826 (Figure 10 and Supplementary Figure S3).

### 2.2. Protein Localisations

To define the individual cellular organisation and tumour activity within the OS aggregoid models, IMC analysis was employed to detect protein and protein modification markers at 1 μm spatial resolution. Proteomic markers relevant to osteosarcoma were selected to identify structural and functional components that influence cancer metabolism.

**Figure 11:**
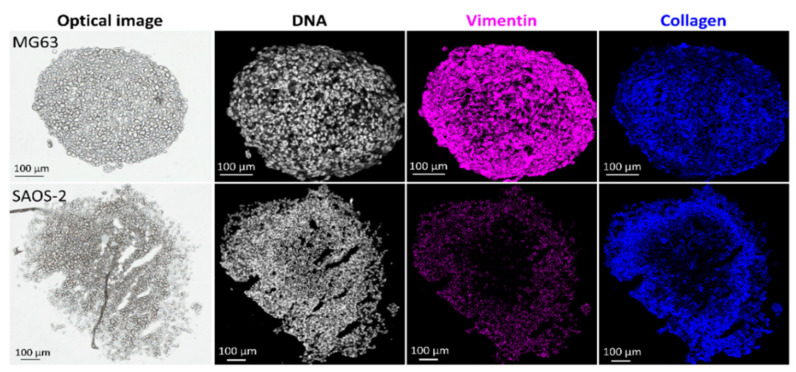
Representative IMC images of protein marker distributions within the OS aggregoid models. Scale bar: 100 μm. DNA intercalator identified individual cells within the aggregoid sections. Protein markers vimentin and collagen expressed identified structural components within the aggregoid tissue.

A generic marker for DNA was used initially to determine the tissue organisation by identifying the nucleus in individual cells within the OS aggregoid models (Figure 11). The differences between the two OS models are defined by the expression of tumour markers, vimentin and collagen. Both structural components show similar co-localisations within the aggregoid sections, yet only expressed within the outer region of the SAOS-2 model as they are more detected across the MG63 aggregoid (Figure 11).

**Figure 12:**
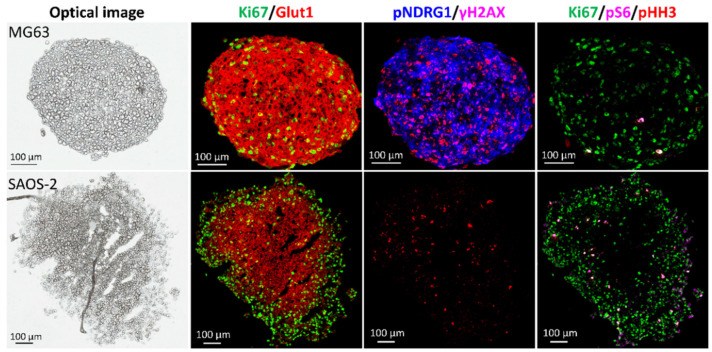
Representative IMC images of protein marker distributions highlight different tumour microenvironment phenotypes within the OS aggregoid models. Scale bar: 100 μm. Protein markers expressed identified the proliferative and hypoxic regions via Ki-67 and Glut1, respectively. Markers in response to DNA damage, pNDRG1 and γH2AX. Proliferative and differentiating phenotypes observed by pS6 and pHH3 markers.

Markers Ki-67, phosphorylated S6 ribosomal protein (pS6) and Histone H3 (pHH3) display differences in the proliferation and differentiation activity between the two OS models (Figure 12). In the SAOS-2 model the expression of Ki-67 is localised within the outer region of the aggregoid, as within the MG63 model the marker is detected throughout the section. This was also observed with the higher expression of pS6 and pHH3 within the outer region of the SAOS-2 aggregoid, yet the limited expression within the MG63 aggregoid was randomly distributed. In addition, a proxy hypoxia marker glucose transporter 1 (Glut1) was detected solely within the core of the SAOS-2 aggregoid as the marker was more homogenously expressed across the MG63 aggregoid (Figure 12). Interestingly the marker for phosphorylated N-myc downregulated gene 1 (pNDRG1) showed significant differences between the two OS models, with high expression in the MG63 aggregoid and no expression in the SAOS-2 aggregoid (Figure 12). 

### 2.3. Elemental Compositions

**Figure 13:**
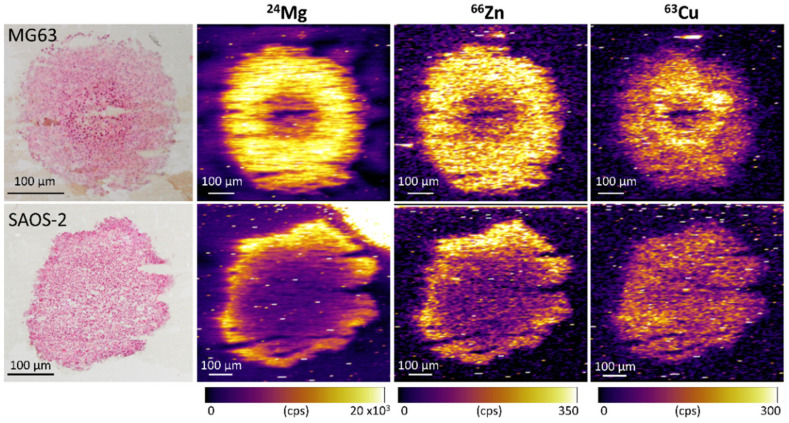
Elemental compositions within the OS aggregoid models identified the tumour microenvironment regions. H&E stain of serial sections of same aggregoid models imaged during LA-ICP-MSI analysis. Scale bar: 100 μm. Elemental maps of ^24^Mg, ^66^Zn and ^63^Cu.

The elemental analysis of the two OS aggregoid models conducted by LA-ICP-MSI, shows similar tumour aggregoid microenvironment phenotypes of an outer annular rim and inner core as previously observed [14]. Slight differences between the metal distributions within the aggregoids were observable. Mg and Zn were co-localised and highly expressed over a large area within the MG63 aggregoid compared to the SAOS-2 aggregoid, which was localised in a thin outer rim (Figure 13). Alternatively, Cu was localised more within the centre of both OS aggregoid models (Figure 13).

## 3. Discussion

### 3.1. OS Metabolite Phenotyping

The different ceramide expression across the models was not unexpected. Ceramides are primarily associated with tumour suppressor activity by triggering anti-proliferative cellular processes such as apoptosis and autophagy [17]. Activation of ceramides are typically induced by cellular stresses such as hypoxia or anti-cancer drug signalling [18,19]. Equally, ceramides have also been associated with cell differentiation and bone development. Hill & Tumber [20] detected ceramides in osteoblasts and determined low levels promoted cellular proliferation, as high levels induced apoptosis to promote bone reformation. From the histology images, a large area of apoptotic bodies was identified in the SAOS-2 model. Thus, the heterogenous ceramide levels within the SAOS-2 aggregoid could be an indicator of apoptotic activity within an inner hypoxic region and cellular differentiation in an outer proliferative zone (Figure 4a). Though, as the ceramide detection within the apoptotic region of HCC827 aggregoids were significantly lower than the SAOS-2 model, it suggests ceramides are more associated with osteoblast-like cells. The discriminatory levels of ceramides between the OS models, however, implies these metabolites differentiate the mature osteoblast phenotype of the SAOS-2 model from the MG63 immature osteoblasts.

In highly proliferating cancer cells, the phospholipid metabolism is altered to promote processes involved in the synthesis of signalling molecules, energy production and the formation of cellular membranes [21]. From the histology image, the MG63 aggregoid displayed a high density of proliferative cells across the tissue and therefore could be reason for the higher detection of such phospholipids. In addition, the role of some specific phospholipids has also been associated with aggressive properties of malignant cancer types [22]. The observation of metabolites PS 38:4 at *m*/*z* 810.530 and PI 38:4 at *m*/*z* 885.549 were significant within the MG63 aggregoids, with some detection in the SAOS-2 aggregoids; PI 38:4 in particular being localised within the periphery of the MG63 aggregoid (Figure 4b) can be rationalised. A previous study comparing the lipidomic profiles of low and high metastatic breast cancer cell lines reported both PS 38:4 and PI 38:4 were markedly greater in the high metastatic cell type [23]. The presence of these lipids could suggest the MG63 aggregoid is exhibiting an aggressive tumoral behaviour, that is if the phospholipids have the same metastatic-promoting effects in OS as they do in breast cancer.

When comparing the metabolite levels within the OS aggregoid models to the OS clinical tissue, the arachidonic acid metabolism was significant (Figure 8). This pathway has been associated with cellular proliferation and differentiation, and the promotion of cancer growth and survival via the cyclooxygenase (COX) pathway [24,25]. This correlates to the observations made in the OS aggregoid models, where proliferative cells were localised within the outer region of the SAOS-2 model, whereas from the histology of the MG63 model, proliferative cells were localised throughout the aggregoid. Therefore, this could suggest the localisation of the fatty acids within the OS human tissue samples could be an indication of highly proliferative activity within the solid tumour regions.

The comparison of the data obtained from the aggregoid models with the patient data showed some marked trends. Interestingly, the two aggregoid models follow a similar detection pattern of a lower FA 18:2, e.g., linoleic acid signal and elevated levels of FA 20:4, e.g., arachidonic acid as OS patient_826 (Figure 8). In contrast, OS patient_882 had higher intensities of FA 18:2. It has been reported that the metabolism of arachidonic acid is also involved in the promotion of angiogenesis, cell invasion and metastasis by the lipoxygenase (LOX) pathway [26]. From the pathology reports, a case of metastasis was reported from OS patient_826 and not in OS patient_882. The observation of an upregulation of FA 20:4, e.g., arachidonic acid, suggests the detection of a possible marker for metastatic potential, primarily within the solid tumour regions of OS patient_826 tissues. In contrast, a higher detection of FA 18:2 within OS patient_882 tissues indicated a slower metabolism to FA 20:4, and thus could identify a lower metastatic potential. The similar detection levels of the fatty acids within the OS aggregoids to the metastatic patient tissue therefore implies the tissue models could be exhibiting metastatic-like behaviour. In addition, the elevated levels of FA 20:4 within the MG63 aggregoid further suggests the cell type forms a higher metastatic-like or aggressive tumour model compared to the SAOS-2 aggregoid.

The phospholipids PS 38:4 at *m*/*z* 810.528 and PI 34:4 at *m*/*z* 885.550 detected significantly in the MG63 aggregoid (discussed earlier) were also detected significantly higher within the metastatic OS patient_826 tissues. The localisations of both species were observed within the large tumorous regions identified by the yellow and sage green cluster of OS patient_826, as low amounts of *m*/*z* 810.528 and *m*/*z* 885.550 were distributed in the orange tumour cluster of OS patient_882 (Figure 9a,b). These specific metabolites could therefore correlate to high metastatic-like activity within OS, as previously observed in highly metastatic breast cancer [23]. This supports the idea of the MG63 aggregoid model displaying a metastatic-like phenotype and demonstrates similarities to the OS patient_826 human tissue. On the other hand, the SAOS-2 samples expressed a lower signal for both phospholipid species and therefore suggests comparisons to a lower metastatic grade OS tissue, such as OS patient_882.

Conversely, the ceramide species that defined the SAOS-2 aggregoid model were significantly expressed within the metastatic OS patient_826 tissues. Cer 34:1; O2 at *m*/*z* 536.505 and its chlorine adduct at *m*/*z* 572.484 [M + Cl]^−^ were localised within the tumorous regions that corresponded mainly to the sage green cluster of OS patient_826 (Figure 10). As discussed, the segmentation analysis highlighted this ROI with tumorous tissue and mineralised bone. The elevated ceramide levels within this region could be indicative of cellular differentiation and osteoid formation within the human tissues [20]. The comparison to the SAOS-2 aggregoid thus suggests the model exhibits similarities to regions in the OS tissue that express the mature differentiating osteoblast phenotype that mediates bone mineralisation. This contrasts with the MG63 model, which is determined as an immature osteoblast phenotype and could be reason to the lack of ceramide detection. Considering the differences in metabolic activity between the two OS aggregoid models and the processes within OS human tissue, it demonstrates how the 3D models can correspond to different phenotypes within the same tumour. This is significant in terms of drug development, as both models can be used to predict the cellular response within heterogeneous tissue.

### 3.2. Protein Localisations Differ between OS Aggregoid Models

From the DNA expression (Figure 11), differences between the MG63 and SAOS-2 sections can be immediately defined. Within the MG63 model, the DNA marker showed whole nuclei expressed homogenously throughout the section. When compared to the SAOS-2 model, a more heterogenous pattern was observed whereby the outer region of the aggregoid showed whole nuclei, as in the core the DNA was more condensed. This is a known indicator of nuclear disassembly and apoptotic bodies [27], and implies a prevalent necrotic region within the SAOS-2 aggregoid compared to the MG63. This observation was consistent across OS aggregoid samples imaged with IMC and agreed with the metabolite data. 

A homogeneous distribution of vimentin and collagen was detected throughout the MG63 aggregoid, as within the SAOS-2 aggregoid the protein markers were prominently expressed within the outer region (Figure 11). Vimentin is known as a fibroblastic intermediate filament and a mesenchymal cell marker in differentiated cell types [10]. A higher expression of vimentin across the MG63 aggregoid compared to the lower expression in the periphery of the SAOS-2 aggregoid could be highlighting the differentiating mesenchymal cells within the models. In addition, an increase in vimentin has been associated with the overexpression of cyclooxygenase enzyme, COX-2 in MG63 cells [28]. COX-2 is involved in the metabolism of arachidonic acid and promotes angiogenesis and migration. Therefore, the vimentin expression complements the metabolite data discussed previously, where significantly high levels of FA 20:4, i.e., arachidonic acid was localised across the MG63 aggregoids, and only observed in the periphery of the SAOS-2 aggregoids.

The localisation of collagen suggests a similar expression as vimentin. Collagen is an important protein in bone when it is mineralised. The more differentiated the OS cells, the more collagen that is produced. Therefore, the levels of collagen could associate with tumour invasion by the nature of the differentiation status of the cells. The localisation of collagen within the periphery of the SAOS-2 aggregoid indicates an area of differentiation within the outer region, as the expression across the MG63 aggregoid suggests differentiated cells are present throughout (Figure 11). One study reported a higher production of collagen type I in SAOS-2 cells in comparison to MG63 cells [29]. The expression of collagen I has also been associated with ALP activity during bone mineralisation [30], an activity that has been detected high in SAOS-2 cells [4]. In contrast, MG63 cells were reported to express higher levels of collagen III than collagen I [31]. Unfortunately, the marker used in this study detected all types of collagen, and therefore the specific type expressed in the aggregoid models was not determined. However, the presence of different collagen types could have influenced the heterogeneity between the models. 

Interestingly, the markers that defined the distinctive proliferative outer region and hypoxic core phenotypes within the lung adenocarcinoma aggregoid [14] are expressed differently between the two OS models. Similar to the HCC827 aggregoid, the SAOS-2 model showed the hypoxia marker, Glut1 localised within centre of the tissue surrounded by an annular expression of the proliferative marker, Ki-67 (Figure 12). This validates the presence of cellular proliferation and differentiation within the periphery of the SAOS-2 model and identifies the core as a region of hypoxia. In the MG63 aggregoid however, a highly intense distribution of Glut1 with an expression of Ki-67 were both detected across the tissue (Figure 12). The purpose of Glut1 is to provide energy through the transport of glucose into cells that is converted into lactate for cancer to grow and survive in a severe microenvironment, such as hypoxia. High levels of Glut1 are therefore heavily associated with hypoxic tumours and has been correlated to metastatic outcome in some patients [32]. In OS, it was reported that the Glut1 gene is overexpressed in correlation with the hypoxia inducible factor (HIF)-1α to promote tumour progression and is predictive of drug resistance and poor outcome in patients [33]. A higher Glut1 expression in MG63 cells compared to SAOS-2 cells has been previously reported [34] and a knockdown of the Glut1 gene showed inhibition of MG63 cell growth [35]. From the imaging data, the expression of Glut1 across the MG63 aggregoid suggests the gene is constantly switched on in all cells, regardless of the microenvironment. This in turn implies there is a constant stability of HIF-1α, consistent with a hypoxic environment but irrespective of oxygen levels in the MG63 model. This could give an indication of an aggressive hypoxic tumour model. This may also explain the Ki-67 expression across the MG63 aggregoid, whereby the cells are able to proliferate under severe hypoxic conditions due to high glucose concentrations imported by the overexpressed Glut1. 

The marker for phosphorylated N-myc downregulated gene 1 (pNDRG1) showed significant differences between the two OS models, with high expression in the MG63 aggregoid and no expression in the SAOS-2 aggregoid (Figure 12). NDRG1 is a stress responsive gene that can have both oncogenic and tumour suppressor roles involved in cellular differentiation, tumour progression and metastasis, hypoxia and DNA damage response [36,37,38]. It was reported an upregulation of NDRG1 was linked to an increase in hypoxia and HIF-1α expression [39]. This could therefore explain the co-localisation of pNDRG1 with Glut1 in the MG63 aggregoid, however no detection was observed in the core of the SAOS-2 aggregoid. Interestingly, the detection of pNDRG1 in the SAOS-2 cell line has been reported [40], however at significantly lower levels than in the MG63 cells. It was thought the decrease in pNDRG1 in the SAOS-2 cells was due to the differentiation state of the cells and the increased invasion potential. The group, however, noted the detection of pNDRG1 had no effect on the invasive properties of MG63 cells and therefore the role of pNDRG1 between the OS cell lines could be due to the differences in differentiation and cell phenotypes. In this study, growing the cells in 3D could have downregulated the NDRG1 gene, hence no detection of the protein marker, however without comparing the expression to 2D cells this cannot be confirmed.

The distribution of pS6 (Figure 12) can be explained as follows. pS6 is an active marker for mTOR signalling which induces cancer growth and is downregulated in the presence of hypoxia [41]. A low expression of the pS6 marker in the MG63 cells is therefore likely associated with the high levels of the Glut1 marker. The reverse expression of these markers is observed in the SAOS-2 model. This is a similar explanation for the pHH3 marker expression which identifies cells undergoing mitosis [42].

### 3.3. Elemental Compositions Complement Protein Expression

The LA-ICP-MSI data (Figure 13) also throws up some interesting observations. Mg is an essential component utilized in many cellular pathways and is particularly required for the cell cycle [43]. Therefore, a lack of Mg can be an indicator non-proliferative activity. From the IMC data, the Ki-67 proliferation marker showed the MG63 model expressed proliferative cells across the tissue, as the SAOS-2 model displayed a distinctive outer proliferative region. The elemental composition of Mg therefore correlated with the Ki-67 expression. In addition to this, it has been reported that Mg ions can promote the differentiation of mesenchymal stem cells to induce growth [44,45]. The elevated levels within the aggregoids could therefore be indicative of the matrix differentiation process in the mesenchymal cells across the tissue.

The absence of Zn in the core of the HCC827 aggregoid has been associated with the activation of HIF-1α in hypoxia as excess Zn was shown to induce proteasomal degradation of HIF-1α [46]. This theory coincides with the proliferative and hypoxic phenotype of the SAOS-2 model. The high Glut1 expression across the whole MG63 aggregoid would however disprove this, as elevated levels of Zn activity were detected. Alternatively, Zn has been linked to vimentin production, whereby the ions influence the assembly of vimentin in tissues [47]. Zn could therefore be highlighting the formation of the structural component within the tissues due to the similar distributions of Zn and vimentin in the MG63 and SAOS-2 models. This would also correlate to the association of Mg with matrix differentiation in the mesenchymal cells.

The detection of Cu ions within the core of the aggregoid model could be due to an upregulation of Cu-efflux transporter, ATP7A induced by the activation of HIF-1α in hypoxia [48]. A large area of Cu was detected within the SAOS-2 model, this is likely due to the large hypoxic region as observed in the H&E stain of the serial section, and in the Glut1 expression in the biological replicate from the IMC data. As for the MG63 H&E stain, it does show a small region of cells within the core that could be experiencing cellular damage, however apoptotic bodies were not present. This would suggest the MG63 aggregoid core is a less dense area due to the natural gradient of nutrients and oxygen and hence this is why Cu is accumulating within this region.

## 4. Materials and Methods

### 4.1. Materials

Alginic acid, CaCl_2_, casein solution, DPX mountant, eosin, EDTA, EtOH, haematoxylin, NaCl, paraformaldehyde (PFA), polyvinylpyrrolidone (PVP), sodium citrate, Triton™ X-100 and xylene substitute were purchased from Sigma-Aldrich (Gillingham, UK). Hydroxypropyl-methylcellulose (HPMC) was purchased from Alfa Aesar (Thermo Fisher Scientific, Heysham, UK).

#### 4.1.1. 3D Cell Culture Growth

MG63 and SAOS-2 cell lines (ATCC) were independently cultured in alpha modified Eagle’s medium (αMEM) (Lonza Ltd., Castleford, UK), and HCC827 cell line was cultured in Dulbecco’s modified Eagle’s medium (DMEM). Media was supplemented with 10% fetal bovine serum (FBS) and 1% penicillin/streptomycin (Lonza Ltd., Manchester, UK). The aggregated 3D cell culture models were generated based on the method of Palubeckaitė et al. [13] and is described as follows: Cells were maintained at 37 °C, 5% CO_2_ and grown to 80% confluence prior to use. To generate the initial tumor spheres, cells were suspended in 1.2% (*w*/*v*) alginic acid (SigmaAldrich, Gillingham, UK) in 0.15 M NaCl at 1 × 10^6^ cells/mL and extruded out of a needle into 0.2 M CaCl_2_ to polymerise the alginate into beads. Beads were washed with 0.15 M NaCl before culturing in DMEM media for 14 days to yield spheroids ∼100 μm in diameter, and media was replaced every 72 h. Alginate beads were dissolved in an alginate buffer (55 mM sodium citrate, 30 mM EDTA, 0.15 M NaCl) to release spheroids into solution. Spheroids were washed with PBS (Lonza, Castleford, UK) before seeding spheroids into 1% agarose-coated 96-well plate in growth medium. Spheroids were cultured for 7 days to form aggregoids of an approximate 1 mm diameter before harvesting. Spheroid and aggregoid development were analyzed by fluorescent staining with Hoechst 33,342 and propidium iodide staining (10 μg/mL each) for 30 min. Fluorescent images were obtained using the Olympus IX81 Microscope (Southend-on-Sea, UK) and images were captured using Cell^F Multifluorescence and Imaging Software (Europa Science Ltd., Cambridge, UK)

#### 4.1.2. Aggregoid Preparation 

Aggregoids were prepared for imaging analysis based on the tissue embedding protocol previously reported [49]. Briefly, aggregoids were washed in PBS prior to embedding in media made of 7.5% HPMC and 2.5% PVP. Embedded tissues were flash frozen in liquid nitrogen and stored at −80 °C. Frozen aggregoids were sectioned at 10 μm thickness using a Leica CM3050 cryostat (Leica Microsystems, Milton Keynes, UK) operating at −18 °C. Sections were thaw-mounted onto polylysine glass slides followed by immediate desiccation with N^2^ and subsequent vacuum packing for storage at −80 °C [50].

#### 4.1.3. Tissue Sample Collection and Handling

Two samples of human bone tissue biopsies which had been previously classified by conventional pathology as osteoblastic osteosarcoma were obtained from the Children’s Cancer and Leukemia Group Tissue Bank. The samples were provided following ethical approval of this study (Project Reference 2017 BS 06). Samples were snap frozen and cryosectioned on a CM1950 cryostat (Leica Biosystems, Milton Keynes, UK). Sections were cut at 10 μm thickness and *n* = 3 sections of each sample from different depths of the tissues were thaw mounted onto positively charged X-tra^®^ adhesive slides (Leica Biosystems, U.K.).

#### 4.1.4. Small Molecule Analysis

Metabolite and lipid images were obtained by DESI-MSI on a Thermo Fisher Q- Exactive mass spectrometer (Thermo Fisher Inc., Offenbach, Germany) at 40 μm spatial resolution as reported in Chapter 2.3.5.3. One technical repeat was acquired with *n* = 3 biological repeats. The DESI-MSI data were analysed by SCiLS™ Lab MVS Premium 3D version 2020a (Bruker Daltonics, Bremen, Germany) RMS normalization. Metabolites and biological pathways were identified by METASPACE (https://metaspace2020.eu, accessed on 6 July 2021). The peak list from each aggregoid section was exported into csv files and grouped together. The data was then imported into MetaboAnalyst 5.0 [51] to conduct multivariate analysis. PCA and PLS-DA were performed on the selected sample groups for each aggregoid model. The samples were normalised by the median, before applying log transformation and Pareto scaling on the data. The data was displayed in scores plots with 95% confidence regions to determine variance between groups. Loadings plots displayed the *m*/*z* values that profiled specific groups. The ion abundances for the *m*/*z* values were generated into histograms for comparison between regions using GraphPad™ Prism^®^ software (La Jolla, CA, USA).

#### 4.1.5. IMC Analysis

Tissues were fixed with 4% PFA for 10 min at RT. Prior to staining, tissues were permeabilized with 1× casein solution containing 0.1% Triton™ X-100 (5 min) at RT. Tissues were then incubated with blocking buffer (1× casein solution) for 30 min at RT. An antibody cocktail (Supplementary Table S1) was made containing the appropriate dilutions for the antibodies. Tissues were incubated with the antibody cocktail overnight at 4 °C. DNA Ir-Intercalator (Fluidigm^®^) was diluted 1:400 and applied to tissues for 30 min at RT. Washes with PBS were performed three times between each step, with the last step washed in deionized water for 30 s. Slides were left to air dry until analysis. Images were acquired on the Hyperion Imaging System (Fluidigm^®^) with the laser tuned to fully ablate the tissue. One technical repeat was acquired with *n* = 1 biological repeat. The imaging data was analysed using the MCD Viewer v1.0.560.2 software (Fluidgim^®^). 

#### 4.1.6. Elemental Analysis

Experiments were conducted using a NexION 350X ICPMS (Perkin Elmer, Waltham, MA, USA) coupled to an UP-213 LA system (New Wave Research, Fremont, CA, USA) with a frequency quintupled 213 nm Nd: YAG laser. Laser parameters were optimised to 6 μm spot size with laser power 46%, 25 μm/s scan speed, 0.07 J cm^−2^ laser fluence, and 20 Hz repetition rate. The sample was ablated line by line with 6 μm raster spacing at 1.31 min acquisition time. For the ICP-MS instrument there was a direct flow with a rate of 1.4 L/min. The following settings were used in standard mode with an 18 L/min plasma gas flow, 1.2 L/min auxiliary gas flow at 1600 W RF power. Isotopes monitored included ^24^Mg, ^66^Zn and ^63^Cu and the instrument was controlled using Syngistix software. One technical repeat was acquired with *n* = 1 biological repeat. Data analysis was achieved using Iolite Software on Igor Pro (WaveMetrics, Tigard, OR, USA). 

#### 4.1.7. Histology Analysis

After DESI-MSI, aggregoid sections were stained using Mayer’s haematoxylin and eosin solutions. Sections were fixed in 4% PFA (10 min) before staining with haematoxylin (1 min). Tissues were rinsed in tap water before and after submerging in acid alcohol. Tissues were subsequently stained with eosin for 30 s prior to washing tap water, then subsequently washed in 3× absolute EtOH (1 min). Finally, tissues were submerged in xylene substitute (1 min) twice and mounted using DPX mountant. Stained tissues were imaged with Aperio CS2 digital pathology scanner (Aperio Tech., Oxford, UK) at 40× and visualized with ImageScope software (Aperio Tech.).

## 5. Conclusions

In this study, a multimodal imaging methodology was employed to characterise two models of osteosarcoma for the purposes of a pre-clinical in vitro tool. Firstly, in-depth phenotyping of the metabolite activity within the two OS aggregoid models was achieved. A comparison of the metabolite data with OS human tissue samples revealed relevant fatty acid and phospholipid markers. Although, annotations of these species require MS/MS analysis for confident identification of the metabolites. From the putative assignments however, it was suggested that the MG63 aggregoids displayed an aggressive tumour model that exhibited metastatic-like potential. Alternatively, the SAOS-2 aggregoids are the more mature osteoblast-like phenotype that expressed characteristics of cellular differentiation and bone development. It was determined the two OS aggregoid models shared similarities of metabolic behaviour with different regions of OS human tissues, specifically of the higher metastatic grade. This is significant in terms of therapeutic research and development to target against aggressive in vivo OS tumours.

In addition, employing IMC showed significant differences in the distribution of protein markers within the OS aggregoid models. The SAOS-2 aggregoid displayed a typical tumour microenvironment with an inner hypoxic core (Glut1 marker) and an outer proliferative region (Ki-67 marker). Interestingly, the MG63 aggregoid protein expression distributions did not show the presence of such distinct regions. Furthermore, the elemental compositions within the aggregoid models corresponded to the protein distributions of proliferative activity and formation of structural components. The complementary nature of both the IMC and LA-ICP-MSI data confirmed the heterogeneity of metabolite distributions between the 3D models.

Overall, multimodal MSI determined the unique characteristics of two OS aggregoid models and improved the understanding of complex tumour microenvironments. MSI analysis of aggregoid models demonstrated a potential methodology to facilitate pre-clinical applications of cancer research and drug development for improved patient outcome.

## Figures and Tables

**Table 1:** Assignments and errors for metabolites detected in specific aggregoid models. Metabolites were filtered by removal of isotope peaks and mass accuracy (<7 ppm). Significant metabolite detection between tumour models was determined by mean intensities. Data is mean (*n* = 3), one-way ANOVA with Tukey post hoc test * *p* < 0.05, ** *p* < 0.01, *** *p* < 0.001.

Metabolite	*m*/*z* (Measured)	Accuracy (ppm Error)	Aggregoid
FA 8:1; O2	173.082	0.0	OS: SAOS-2 *
FA 9:1; O2	187.098	0.0	All
FA 11:1; O	199.136	0.0	All
FA 17:1	267.232	0.0	All: HCC827 *
FA 18:5	273.186	1.0	All
FA 18:3	277.217	−0.4	OS
FA 18:2	279.235	6.3	All: OS
FA 18:1	281.248	−0.4	All: HCC827 *
FA 18:2; O	295.229	3.4	All: HCC827 *
FA 20:5	301.218	0.0	All: MG63 *
FA 20:4	303.231	−0.2	All: MG63 *
FOH 27:0	395.427	3.3	MG63 *
FA 20:4	457.371	5.0	SAOS-2 **
Cer 32:1; O2	508.474	1.1	SAOS-2 ***
FAHFA 34:2; O	535.472	−0.9	All: HCC827 *
Cer 34:1; O2	536.506	1.9	SAOS-2 ***
Cer 32:1; O2 [M + Cl]^−^	544.449	−3.2	OS: SAOS-2 ***
FAHFA 36:2; O	563.504	−1.0	All: HCC827 ***
Cer 34:1; O2 [M + Cl]^−^	572.480	−2.2	OS: SAOS-2 ***
PI 18:0	599.320	−0.2	MG63 *
PE O-28:1	618.450	−1.0	SAOS-2 **
PE O-30:1	646.482	−0.3	SAOS-2 ***
PE O-34:2	700.527	−2.1	OS: MG63 ***
PE 34:1	716.524	0.1	OS: MG63 *
PE O-36:6	720.498	1.1	MG63 **
PE O-36:2	728.562	2.5	OS ***
PC O-33:1	730.577	1.7	OS: SAOS-2 *
PS O-33:0	734.533	−1.2	MG63
PC 33:2	742.540	1.4	MG63 **
PG 34:1	747.516	−3.0	OS: MG63 *
PE O-38:5	750.544	−0.3	MG63 *
PS 34:1	760.518	7.0	OS *
PE O-38:6; O	764.526	2.7	MG63 **
PS 36:1	788.544	−0.4	OS: MG63 **
PS 38:4	810.530	1.3	MG63 **
PG 40:7	819.519	0.7	MG63 **
PS 40:6	834.523	−6.3	OS *
PI 38:4	885.549	−0.9	OS: MG63 *

**Table 2:** Assignments and errors for metabolites detected in both the OS human tissue samples and OS aggregoid models, MG63 and SAOS-2. Metabolites were filtered by removal of isotope peaks and mass accuracy (<7 ppm). Significant metabolite detection between tumour models was determined by mean intensities. Data are mean (*n* = 3), unpaired *t* test * *p* < 0.05, ** *p* < 0.01, *** *p* < 0.001, **** *p* < 0.0001.

Metabolite	Osteosarcoma Tissue				Aggrecoid	
	* m/z * (Measured)	Accuracy (ppm Error)	Patient Sample	* m/z * (Measured)	Accuracy (ppm Error)	Aggregoid Model
FA 18:2	279.234	2.4	Both	279.235	6.3	Both
FA 18:1	281.247	−0.4	Both	281.248	– 0.4	Both
FA 18:2;O	295.229	2.9	OS_882 *	295.229	3.4	MG63 *
FA 18:1;O	297.242	−6.0	Both	297.242	– 5.9	MG63 *
FA 20:4	303.231	0.2	OS_826 ***	303.231	– 0.2	Both
FA 18:2;O2	311.224	3.5	OS_882 *	311.223	0.7	Both
Cer 32:1;O2	508.474	0.7	OS_826 **	508.474	1.1	SAOS-2 ***
FAHFA 34:2;O	535.473	1.1	Both	535.472	–0.9	Both
Cer 34:1;O2	536.505	0.0	OS_826 ***	536.506	1.9	SAOS-2 ***
Cer 32:1;O2 [M+Cl]^-^	544.451	0.6	OS_826 **	544.448	–3.2	SAOS-2 ***
FAHFA 36:2;O	563.505	0.1	Both	563.504	–1.0	Both
FAHFA 36:1;O	565.522	4.2	Both	565.522	2.9	Both
Cer 34:1;O2 [M+CI]^-^	572.484	4.2	OS_826 ***	572.480	–2.2	SAOS-2 ***
PE O-28:1	618.451	1.6	OS_826 **	618.450	–1.0	SAOS-2 **
PE O-30:1	646.613	−2.1	OS_826 ***	646.615	1.0	Both
Cer 40:1;O2 [M+CI]^-^	656.578	3.5	OS_826 **	656.576	0.2	Both
PA 36:3	701.514	3.0	Both	701.511	–1.0	Both
PE 34:1	716.524	0.3	OS_826 *	716.524	0.1	MG63 *
PE O-36:2	728.560	−0.4	OS_826 *	728.562	2.5	Both
PC O-33:1	730.575	−0.8	Both	730.577	1.7	SAOS-2 *
PC 33:2	742.539	0.1	OS_826 *	742.540	1.4	MG63 *
PG 34:1	747.517	−1.1	OS_826 **	747.516	–3.0	MG63 *
PE O-38:5	750.542	−2.6	OS_826 *	750.544	–0.3	MG63 *
PE O-38:6;O	764.524	0.4	OS_826 *	764.526	2.7	MG63 **
PS 36:1	788.544	−1.3	OS_826 **	788.544	–0.4	MG63 **
PS 38:4	810.528	−1.5	OS_826 **	810.530	1.3	MG63 *
PS 40:4	838.559	−1.3	OS_826 **	838.562	2.2	Both
PI 38:4	885.550	0.5	OS_826 **	885.549	–0.9	MG63

## Data Availability

The data obtained during this project will be made available at the Sheffield Hallam University Research Data Archive SHURDA https://shurda.shu.ac.uk/.

## Supplementary Materials

The following are available online at https://www.mdpi.com/article/10.3390/metabo11080506/s1, Figure S1: Mean intensity plotted on bar graph of fatty acid detection within OS human tissue and OS aggregoid models, Figure S2: Mean intensity plotted on bar graph of metastasis-related phospholipid species within OS human tissue and OS aggregoid models, Figure S3: Mean intensity plotted on bar graph of ceramide species detected within OS human tissue and OS aggregoid models, Table S1: Protein and protein modification markers and the respective antibodies used.
