# Supplementary material for: Comparison of Osteosarcoma Aggregated Tumour Models with Human Tissue by Multimodal Mass Spectrometry Imaging
Source: Metabolites. 2021 Jul 31;11(8):506. doi: 10.3390/metabo11080506 (PMC8401535; doi:10.3390/metabo11080506)
Supplement: Supplementary file 1 [file metabolites-11-00506-s001.zip › metabolites-1309752-supplementary.pdf]

Supplementary Information

Supplementary Figure S1. Mean intensity plotted on bar graph of fatty acid detection within OS human tissue and OS aggregoid models. Data is mean ± SD (n=3), unpaired t-test * $p < 0.05$, ** $p < 0.01$. Peaks identified in human tissue a) *m/z* 279.234, FA 18:2; b) *m/z* 281.247, FA 18:1; c) *m/z* 303.231, FA 20:4. Peaks identified in OS models d) *m/z* 279.235, FA 18:2; e) *m/z* 281.248, FA 18:1; f) *m/z* 303.231, FA 20:4.

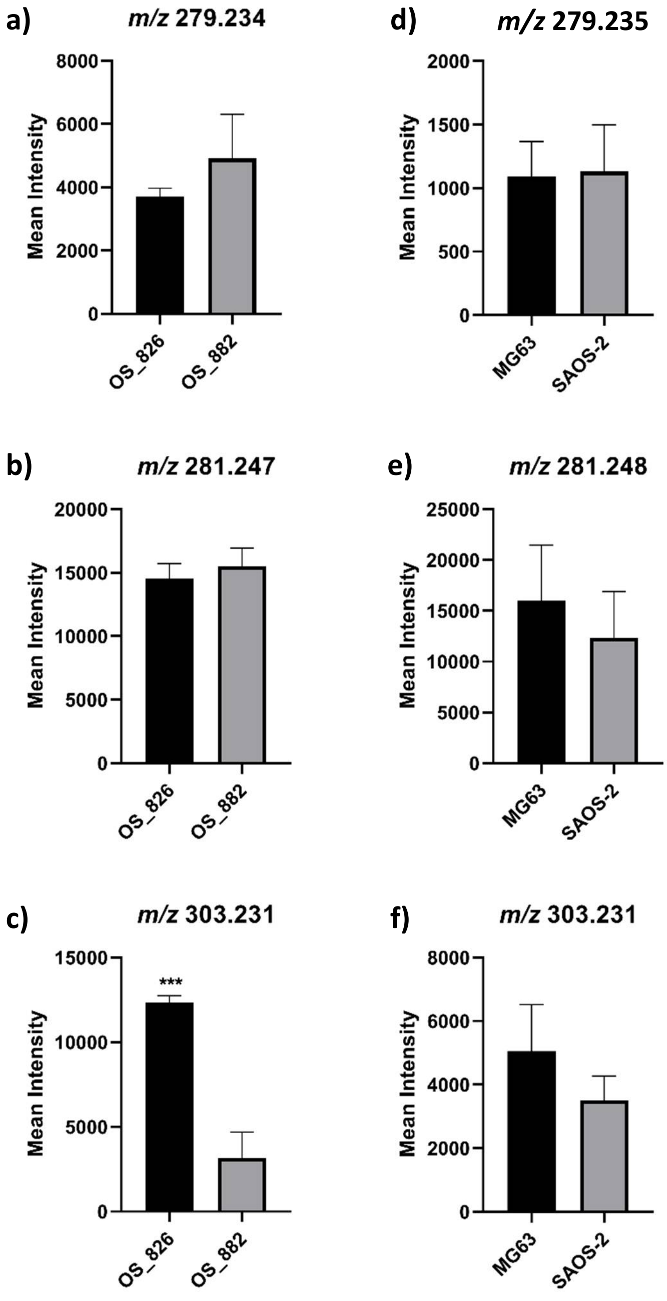

Supplementary Figure S2. Mean intensity plotted on bar graph of metastasis-related phospholipid species within OS human tissue and OS aggregoid models. Data is mean ± SD (n=3), unpaired t-test * $p < 0.05$, ** $p < 0.01$. Peaks identified in human tissue a) *m/z* 810.528, PS 38:4; b) *m/z* 885.550, PI 38:4. Peaks identified in OS models d) *m/z* 810.530, PS 38:4; e) *m/z* 885.549, PI 38:4.

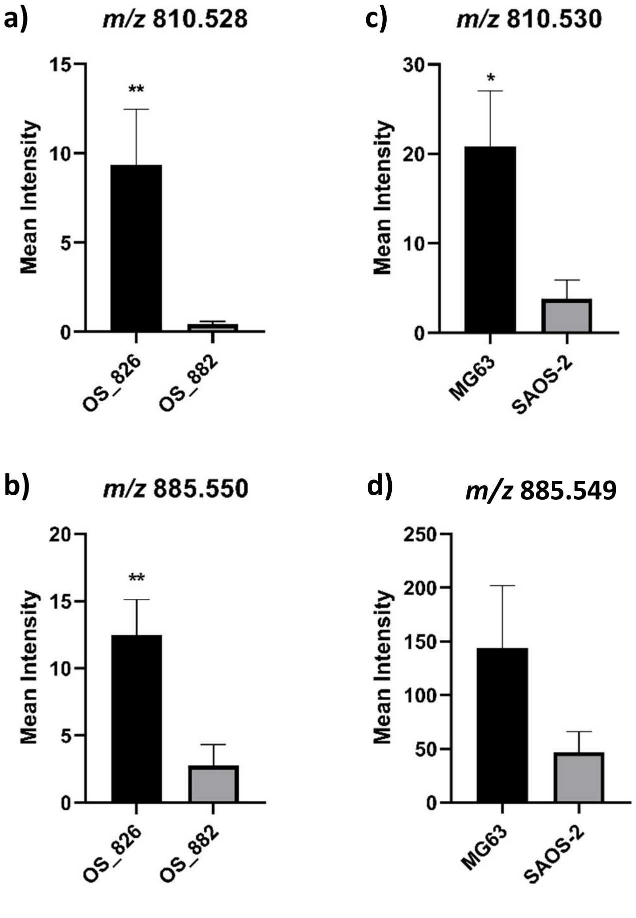

Supplementary Figure S3. Mean intensity plotted on bar graph of ceramide species detected within OS human tissue and OS aggregoid models. Data is mean ± SD (n=3), unpaired t-test * p < 0.05, ** p < 0.01, *** p < 0.001. Peaks identified is human tissue a) *m/z* 536.505, Cer 34:1;O2; b) *m/z* 572.484, Cer 34:1;O2 [M+Cl]$^{-}$. Peaks identified in OS models c) *m/z* 536.504, Cer 34:1;O2; d) *m/z* 572.480, Cer 34:1;O2 [M +Cl]$^{-}$.

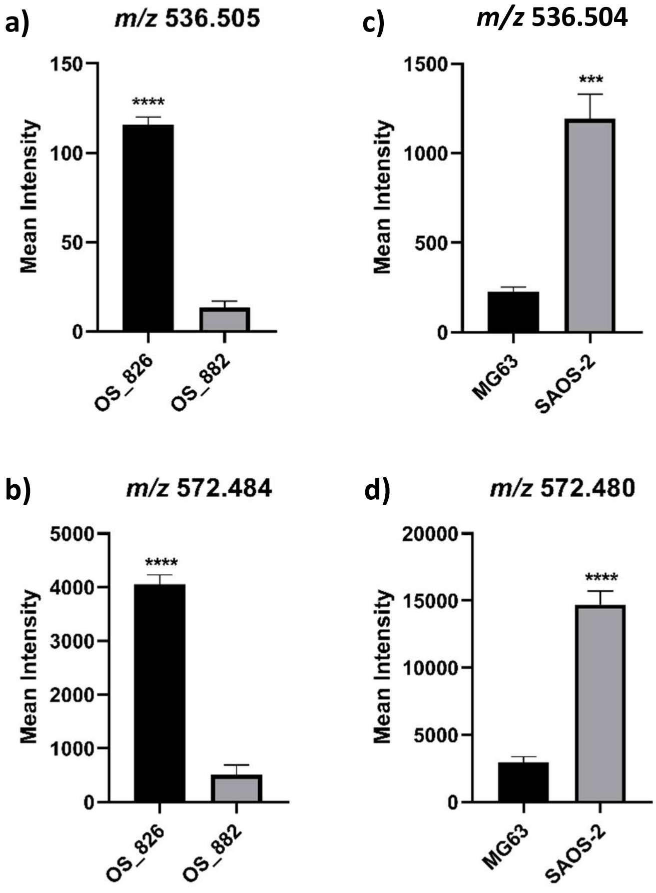

Supplementary Table S1. Protein and protein modification markers and the respective antibodies used. Antibodies were metal-tagged and validated in house by AstraZeneca.

Target	Annotation	Clone	Metal tag	Host specimen	Product code
Vimentin	Mesenchyl	D21H3	143Nd	Rabbit	3143027D
pNDRG1	Stress response	D98G11	159Tb	Rabbit	5282
GLUT1	Proxy hypoxia marker	EPR3915	160Gd	Rabbit	ab196357
Ki67	Proliferation	B56	162Dy	Mouse	3162012B
Collagen	Extracellular matrix	Polyclonal	169Tm	Goat	3169023D
pS6	mTOR signalling	N7-548	172Yb	Mouse	3172008A
yH2AX	DNA damage	JBW301	173Yb	Mouse	05-636
pHH3	Mitosis	HTA28	176Yb	Rat	3176024D
